# Initial experimentation with tobacco is associated with subsequent tobacco use patterns among youth in the United States

**DOI:** 10.1371/journal.pone.0308964

**Published:** 2024-09-27

**Authors:** Omar El-Shahawy, Kandi L. Walker, Allison M. Groom, Thomas J. Payne, Lindsay K. Tompkins, Anshula Kesh, Robyn Landry, Jack Pfeiffer, Aida L. Giachello, Thanh-Huyen T. Vu, Jennie Z. Ma, Rose Marie Robertson, Sasidhar Gunturu, Michael J. Blaha, Joy L. Hart

**Affiliations:** 1 American Heart Association Tobacco Center for Regulatory Science, Dallas, Texas, United States of America; 2 New York University Grossman School of Medicine, New York, New York, United States of America; 3 University of Louisville, Louisville, Kentucky, United States of America; 4 American Heart Association, Dallas, Texas, United States of America; 5 University of Mississippi Medical Center, Jackson, Mississippi, United States of America; 6 Northwestern University Feinberg School of Medicine, Evanston, Illinois, United States of America; 7 University of Virginia, Charlottesville, Virginia, United States of America; 8 Department of Psychiatry, BronxCare Health System, Bronx, New York, United States of America; 9 Department of Psychiatry, Icahn School of Mount Sinai, New York, New York, United States of America; 10 Johns Hopkins Ciccarone Center for the Prevention of Cardiovascular Disease, Baltimore, Maryland, United States of America; NYU Grossman School of Medicine: New York University School of Medicine, UNITED STATES OF AMERICA

## Abstract

Understanding the association between initial experimentation with a tobacco product and subsequent patterns of tobacco use among youth is important to informing prevention activities for youth in the US. We conducted an online survey from August to October 2017 among youth aged 13–18 years. The current analysis focused on respondents reporting initial experimentation with any tobacco product (n = 2,022). Using multinomial logistic regression, we examined the association between first tobacco product tried (cigarettes; cigars including cigarillos, little cigars, and bidis; electronic nicotine delivery systems (ENDS); smokeless and chewing tobacco; or hookah) with subsequent patterns of tobacco use while adjusting for covariates. Of the youth who experimented, 56.8% were non-current tobacco users. Of current tobacco users (n = 934), 13% were exclusive ENDS users, 5.3% exclusive combustible mono-users, 13.4% ENDS plus combustible poly-users, 3.3% combustible product only poly-users, and 8.2% other tobacco poly-users. The most common type of first tobacco product tried was ENDS (44.7%), followed by cigarettes (35.0%) and cigars (8.6%). Those who experimented with combustible tobacco products were less likely to be exclusive ENDS users [Relative Risk Ratio (RRR) = 0.46; 95% CI = 0.28, 0.73 for cigarettes; RRR = 0.32; 95% CI = 0.13, 0.81 for cigars; and RRR = 0.33; 95% CI = 0.14, 0.79 for hookah] when compared to non-current tobacco users (reference group). Tobacco product choices for initial experimentation appear to play a role in subsequent tobacco use patterns among youth. Understanding the reasons behind initial product choice may inform our understanding regarding the reasons for subsequent current tobacco product use, thus informing youth prevention efforts.

## Introduction

Cigarette smoking is the leading cause of premature death in the United States [1]. Despite the documented negative health effects of use, the tobacco control landscape faces an unprecedented diversity of tobacco and nicotine-containing products [2, 3]. Beyond combustible cigarettes, these products include pipe and hookah, cigars, cigarillos, little cigars, bidis, smokeless tobacco, and newer products, such as electronic nicotine delivery systems (ENDS) and heat-not-burn options. ENDS are currently regulated as a tobacco product under the Food and Drug Administration’s deeming rule [4]. Non-cigarette tobacco products, particularly ENDS, are often perceived as safer than cigarettes and may appeal to individuals, including those youth who have traditionally been considered lower risk for initiating tobacco product use [5, 6]. These changes in the tobacco market coincide with alarming reports of increasing poly use of combustible and non-combustible tobacco products, which are also associated with increases in risky behavior among youth and lower likelihood of quitting tobacco use [6–10].

There is an ongoing debate among tobacco control advocates regarding the potential association of the initial experimentation with various tobacco products and patterns of subsequent youth tobacco use [11]. However, there is limited understanding of whether initiation with a specific tobacco product, including ENDS, increases the odds of using other tobacco products or poly-tobacco use [10, 11]. From a population health perspective, several tobacco control researchers conceptualize that the harm from tobacco use can be viewed from a continuum of risk perspective. On this continuum, ENDS and smokeless tobacco (i.e., non-combustible) are considered less harmful than combustible tobacco [12]. However, recent evidence suggests ENDS use attenuates attempts to quit, maintains addiction to nicotine, and contributes to the development of specific health problems such as cardiovascular disease [13] or vaping-related pulmonary illness [14–16]. There also may be differences in youth perceptions that are associated with the choice of product and ultimately their pattern of use [6]. Additionally, the factors associated with patterns of product use have implications for different health campaigns based on the class of products used (e.g., targeting smokeless tobacco products is unrelated to secondhand smoking effects).

Understanding the association of initial experimentation with a tobacco product and subsequent patterns of tobacco use among youth is important to informing primary and secondary prevention activities for youth in the US. Thus, the purpose of this study is to assess the association between initial experimentation and subsequent tobacco use among a nationally representative sample of US youth. Based on prior research, [7–10, 17–22] we hypothesize that initial experimentation with a particular tobacco product will be associated with youth subsequent tobacco use patterns. For example, experimentation with non-combustible products like ENDS will be associated with the likelihood of continued use of the same product class rather than using other tobacco products (e.g., combustible tobacco).

## Materials and methods

### Study population

From August to October 2017, researchers conducted an online survey of US youth aged 13–18. This approach provided the ability to reach a diverse, national sample, recruited by an established marketing research vendor specializing in youth. The vendor manages an online panel of 65,000 US youth and young adults born between 1982 and 2004. Members are recruited via buzz campaigns, newspaper ads, and social networks. Panelists earn points for each completed survey and can redeem the points for prizes. Panel management procedures comply with marketing research industry standards set by professional marketing research associations, including appropriate disclosure of any potential conflicts of interests that may interfere with research. Quotas were set for key demographics, allowing sampling based on following factors: age, sex, and race/ethnicity. Non-Hispanic Blacks and Hispanics were oversampled to ensure sufficient sample sizes for comparison by race and ethnicity. The data were weighted from an overall population perspective to be representative of the US population in terms of sex, age, race, ethnicity, and geographic region. Procedures for obtaining proper online consent were implemented. No identifying information was collected, and guidelines established by the Children’s Online Privacy Protection Act (COPPA) were followed. Youth participants were given assent forms and could elect not to participate. Parental consent was obtained for teen panelists under the age of 18. The study team had no direct contact with recruited individuals. The Chesapeake Institutional Review Board reviewed and approved this study.

The study initial recruitment sample consisted of 3,174 participants (3,000 after weighting). The sample was weighted against the 2017 US Census, and weighting variables were less than one, thus reducing the sample size. Two groups were recruited and included in the main analytical dataset after accounting for missing observations: (a) ENDS Users, defined as youth who have ever tried ENDS (n = 1617), including a subset of Dual Users within this group (n = 1227, who have tried ENDS and another tobacco product) and (b) a Control Group, defined as youth who have never tried ENDS (n = 1037). The current analysis focused on respondents who reported initial experimentation with any tobacco product type from the overall sample (n = 2,022 unweighted/1,673 weighted). Initial experimentation was determined by respondent reports of ever trying any tobacco product, including ENDS.

### Tobacco experimentation and demographic characteristics

We asked participants to indicate the first tobacco product tried—cigarettes; cigars, including cigarillos, little cigars, and bidis; ENDS; smokeless dip and chewing tobacco; or hookah. This question allowed only one response and therefore responses are mutually exclusive. We characterized demographics as follows: age (13–14; 15–16; or 17–18 years), sex (female or male), sexual orientation (heterosexual or LGB+ [Lesbian, Gay, Bisexual or other]), race/ethnicity (Hispanic, Non-Hispanic [NH] White, NH Black, or other), residential area (urban, suburban, or rural), and receiving public assistance (yes, no, or not sure). Receiving public assistance was assessed with the question “Do you/your family receive government public assistance (such as Medicaid, Section 8 for housing assistance, Obama phone, food stamps/the link card/SNAP, or other government financial help) or any non-government assistance (e.g., religious institutions/charitable organizations)?”.

### Primary outcome

The current tobacco use pattern was the primary outcome measure. Current use status was defined as use of any tobacco product either daily, weekly or at least monthly; and then further stratified into six mutually exclusive categories: a) Non-current users (reported not using any product now or using them less than monthly), b) Exclusive ENDS users (reported using only ENDS and no other tobacco product), c) Exclusive combustible mono-users (reported using only one of the following combustible products, cigarettes, hookah, cigars, cigarillos, little cigars, or bidis and no other tobacco project); d) ENDS plus combustible tobacco poly-users (reported using ENDS in addition to at least one combustible tobacco product), e) Combustible only poly-users (reported using two or more combustible tobacco products), and f) Other tobacco poly-users (reported using other possible combinations, e.g., ENDS and chewing tobacco; combustible and smokeless tobacco). We also included exclusive smokeless dip and chewing tobacco with the other tobacco users group given their low prevalence among the sample precluding including them as a separate group; chewing tobacco (n = 2) and smokeless dip (n = 15).

### Statistical analyses

All statistical analyses were performed using Stata version 14.0 (StataCorp, College Station, TX) with the “svy” command, which was appropriate to account for weighting. Missing responses were considered to indicate non-users, likely rendering estimates of prevalence conservative. Percentages are weighted to ensure representativeness.

Demographic characteristics and initial experimentation with tobacco products are presented as weighted percentages with 95% confidence intervals within each outcome group (non-current users, exclusive ENDS users, exclusive combustible mono-users, ENDS plus combustible poly-users, combustible only poly-users, and other tobacco poly-users). Using multinomial logistic regression modeling, we examined the association of the first tobacco product tried with subsequent current patterns of tobacco use, while adjusting for sex, sexual orientation, age, race/ethnicity, residential area, and public assistance as a proxy for socio-economic status. The results are reported as adjusted Relative Risk Ratios (RRRs) with 95% confidence intervals (CIs), employing a significance level of p<0.05.

## Results

**Fig 1:**
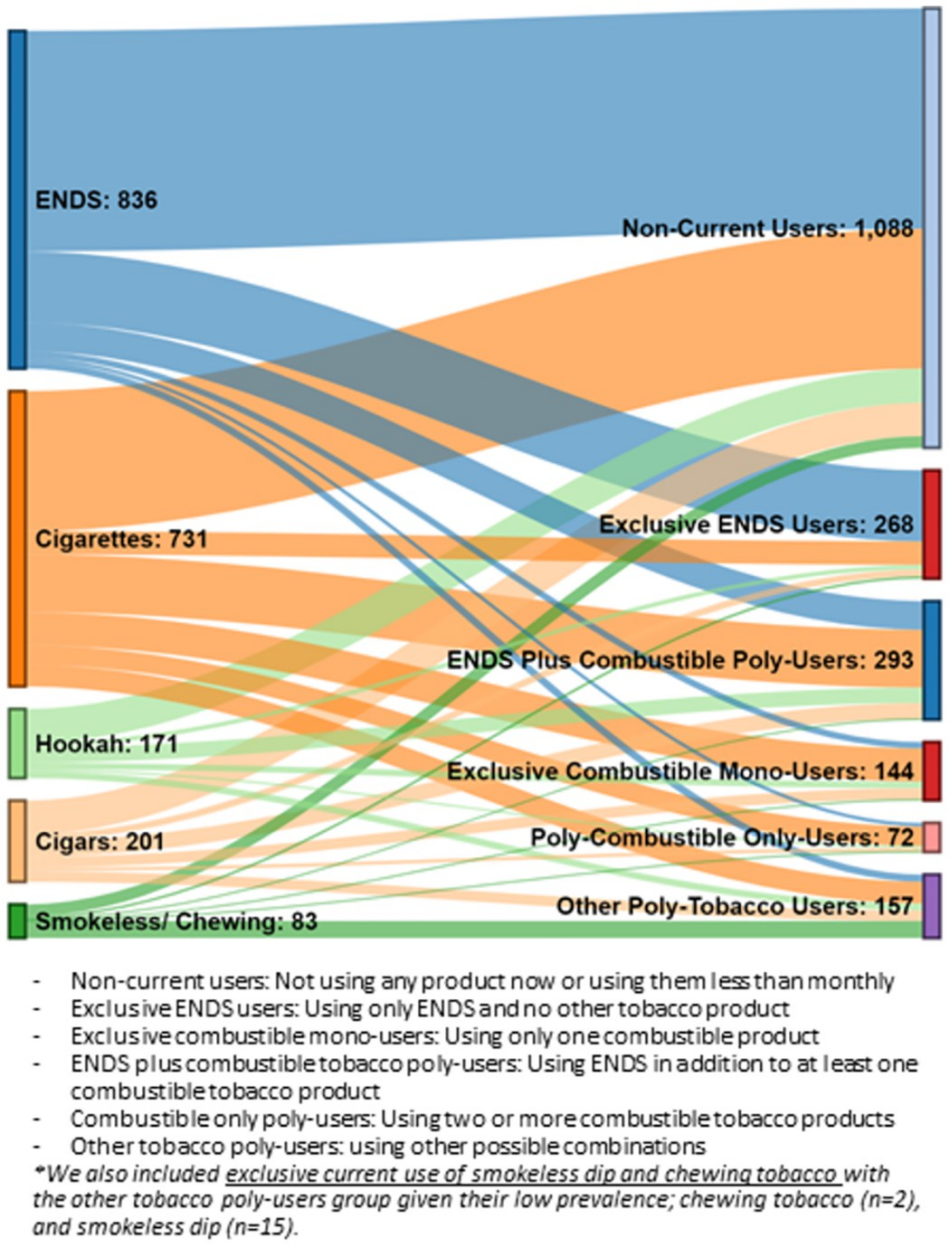
Initial experimentation by tobacco product and subsequent current tobacco use pattern.

Overall, 45.2% were female; 76.8% were heterosexual; 50.9% were aged ≥ 17 years old; 62.5% were NH White; and 22.2% were Hispanic (see Table 1). Among our sample, the most common initial tobacco product tried was ENDS (44.7%), followed by cigarettes (35.0%) and cigars (8.6%). Nearly half of the respondents (43.2%) progressed from experimentation to subsequent tobacco use. Among the youth who first experimented with ENDS (n = 836), one-third (32.8%) continued to be subsequent tobacco users, which is considerably lower than the proportion that continued to subsequent use after experimenting with other products. Approximately half of the youth who first experimented with other products continued to be subsequent tobacco users (47.4% for cigarettes, 61.7% for cigar group [cigars, cigarillos, or little cigars], 59.9% for smokeless dip and chewing tobacco, and 54% for hookah). Further Fig 1 illustrates the tobacco experimentation groups and their subsequent tobacco use pattern. The flow from experimentation to current use behavior shows that a higher proportion of initial ENDS experimenters were current non-users than those who had initially experimented with other combustible tobacco products. Among current users (n = 934), more than half (56%) were poly-tobacco users. More specifically, 31% were ENDS plus combustible poly-users, 8% were combustible only poly-users, and 17% were other tobacco poly-users. Exclusive use accounted for 44% of the sample with 29% exclusive ENDS users and 15% exclusive combustible users (i.e., using only one combustible product).

**Table 1:** Sample characteristics and tobacco use initial experimentation history among youth in the United States (2017).

	Total Sample (N^a^ = 2,022)	Non-current users (n^a^ = 1,088) 56.8%^b^	Current Tobacco Use (n = 934, 42.2% ^b^)					
			Exclusive ENDS users (n^a^ = 268) 13%^b^	Exclusive combustible mono-users (n^a^ = 144) 5.3%^b^	ENDS plus combustible poly-users (n^a^ = 293) 13.4%^b^	Poly combustible only (n^a^ = 72) 3.3%^b^	Other poly tobacco users^c^ (n^a^ = 157) 8.2%^b^	p-value^d^
	N^a^ (%^b^)	% (95% CI)^b^	% (95% CI)^b^	% (95% CI)^b^	% (95% CI)^b^	% (95% CI)^b^	% (95% CI)^b^	
**First tobacco tried**								< .001
ENDS	836 (44.7)	52.9 (48.8, 57.0)	71.8 (63.7, 78.7)	11.0 (04.7, 23.2)	26.9 (20.4, 34.6)	15.9 (06.6, 33.4)	7.7 (03.8, 15.1)	
Cigarettes	731 (35.0)	32.4 (28.7, 36.4)	19.7 (13.9, 27.2)	56.6 (42.5, 69.7)	45.1 (37.4, 53.0)	60.9 (43.0, 76.3)	36.3 (26.8, 47.0)	
Cigar Group	201 (08.6)	5.8 (04.2, 08.0)	2.7 (1.2, 6.0)	24.0 (13.1, 39.7)	15.0 (10.3, 21.4)	11.0 (03.9, 27.2)	16.3 (10.4, 24.7)	
Smokeless or Chewing	83 (05.2)	3.7 (02.3, 05.9)	3.6 (1.3, 9.7)	0.3 (00.1, 00.9)	0.2 (0.04, 01.3)	4.3 (00.6, 24.7)	30.4 (21.7, 40.8)	
Hookah	171 (06.4)	5.2 (03.7, 07.2)	2.2 (1.0, 4.5)	8.3 (03.7, 17.4)	12.7 (08.3, 19.0)	8.0 (02.2, 24.7)	9.3 (04.8, 17.2)	
**Sex:** Female	1,082 (45.2)	54.6 (50.5, 58.6)	37.6 (29.5, 46.3)	57.0 (43.2, 69.8)	28.6 (22.5, 35.6)	56.4 (38.8, 72.5)	7.6 (03.9, 14.3)	< .001
**Sexual orientation:** LGB+	444 (23.2)	25.0 (21.5, 28.7)	20.8 (14.4, 29.1)	25.8 (15.9, 38.9)	21.9 (16.3, 28.7)	26.9 (14.9, 43.8)	14.0 (07.9, 23.7)	0.036
**Age**								< .001
13–14	211 (14.7)	15.9 (12.9, 19.5)	13.3 (07.8, 21.7)	19.8 (09.0, 38.1)	8.0 (04.7, 13.2)	19.6 (07.9, 40.9)	14.6 (09.6, 21.4)	
15–16	491 (34.4)	38.0 (34.1, 42.0)	33.1 (25.8, 41.3)	14.9 (08.0, 26.3)	29.5 (23.1, 36.8)	15.4 (06.5, 32.1)	39.5 (30.1, 49.7)	
17–18	1,320 (50.9)	46.1 (42.1, 50.2)	53.7 (45.0, 62.2)	65.2 (49.7, 78.1)	62.5 (54.9, 69.6)	65.0 (46.0, 80.2)	45.9 (35.6, 56.5)	
**Race/ethnicity**								< .001
NH-White	1,180 (62.5)	59.5 (55.4, 63.5)	69.6 (60.8, 77.2)	63.1 (49.6, 74.8)	62.3 (54.2, 68.8)	68.0 (49.8, 82.0)	67.5 (58.4, 78.8)	
NH-Black	251 (11.7)	13.6 (11.2, 16.5)	6.3 (03.8, 10.4)	20.9 (12.8, 32.1)	10.9 (07.1, 16.2)	7.4 (01.8, 25.4)	4.9 (02.1, 10.8)	
Hispanic	403 (22.2)	23.2 (19.7, 27.2)	19.9 (13.1, 29.1)	15.1 (07.3, 28.6)	23.3 (16.6, 31.8)	21.3 (10.2, 39.3)	22.3 (13.9, 33.9)	
Other	188 (03.6)	3.7 (02.8, 04.9)	4.2 (02.3, 07.5)	0.94 (00.2, 03.9)	4.1 (02.2, 07.5)	3.3 (00.6, 16.8)	3.3 (01.3, 07.9)	
**Residence area**								0.001
Urban	765 (36.8)	35.9 (32.1, 40.0)	33.0 (25.5, 41.3)	42.5 (30.1, 56.0)	38.9 (31.5, 46.8)	42.5 (26.7, 60.1)	39.8 (30.5, 49.8)	
Suburban	792 (39.1)	39.8 (35.9, 43.9)	44.8 (36.4, 53.6)	23.1 (12.3, 39.2)	46.7 (38.9, 54.6)	24.7 (13.5, 40.8)	28.8 (20.1, 39.4)	
Rural	465 (24.0)	24.2 (20.9, 27.9)	22.2 (15.6, 30.7)	34.8 (23.0, 48.7)	14.4 (09.8, 20.6)	32.7 (18.4, 51.3)	31.5 (22.2, 42.5)	
**Public Assistance**								< .001
Yes	647 (30.5)	28.7 (25.1, 32.6)	23.3 (16.8, 31.4)	44.4 (31.6, 57.9)	28.4 (22.1, 35.8)	44.3 (28.4, 61.6)	43.4 (33.6, 53.7)	
No	1,158 (57.4)	55.3 (51.2, 59.4)	68.4 (59.9, 75.8)	48.0 (34.6, 61.7)	63.1 (55.4, 70.2)	49.1 (32.4, 66.0)	54.7 (44.4, 64.6)	
Not Sure	217 (12.1)	16.0 (13.2, 19.3)	8.3 (04.8, 14.1)	7.6 (03.3, 16.8)	8.4 (05.0, 13.8)	6.6 (01.8, 21.8)	1.9 (00.6, 05.3)	

ENDS: Electronic Nicotine Delivery Systems; Cigar Group: cigars, cigarillos, little cigars and bidis

^a^Unweighted;

^b^Weighted percentages;

^c^Other poly tobacco users include all other current tobacco use combinations including combustible and/or non-combustible products, in addition to exclusive smokeless and chewing users (n = 17);

^d^Chi-square test p-value

Significant differences in sex, age, sexual orientation, residential area, public assistance, and first tobacco product tried were found between current tobacco use groups. For example, more exclusive ENDS and ENDS plus combustible tobacco users were male and reported that their families did not receive public assistance. More exclusive ENDS users (71.8%) reported that ENDS were the first type of product used, but cigarette experimenters who continued to use tobacco were mostly exclusive combustible mono-users, ENDS plus combustible poly-users or poly combustible only users (56.6%, 45.1%, 60.9%, respectively, from each use group). In addition, more ENDS plus combustible poly-users (12.7%) reported that hookah was the first type of tobacco used, compared to all other groups (Range: 2.2–9.3%; see Table 1).

Table 2 presents the association of subsequent tobacco use pattern with first tobacco product tried. Those who first tried cigarettes, cigars, or hookah compared to ENDS were less likely to be exclusive ENDS users [Relative Risk Ratio (RRR) = 0.46, 95% CI = 0.28, 0.73; RRR = 0.32, 95% CI = 0.13, 0.81; RRR = 0.33, 95%CI = 0.14, 0.79, respectively]. The relative risk of combustible mono-use was highest among the cigar group experimenters (RRR = 17.3, 95% CI = 5.04, 59.34), followed by hookah (RRR = 7.30; 95% CI = 2.04, 26.15) and cigarettes (RRR = 6.90; 95% CI = 2.62, 18.19). The least risk to be an exclusive combustible or an ENDS plus combustible current user was among the smokeless dip and chewing tobacco experimenters (RRR = 0.18, 95% CI = 0.04, 0.91; RRR = 0.10, 95% CI = 0.01, 0.66, respectively). Those who first experimented with cigarettes, in comparison to ENDS, were consistently more likely to belong to one of the combustible tobacco product groups (RRR range: 2.95–8.63; p < .001). In contrast, compared to non-current tobacco users, those who were currently mono-combustible tobacco product users only were more likely to have first experimented with a combustible tobacco product in comparison to ENDS (RRR = 6.90; 95% CI = 2.62, 18.19 for cigarettes; RRR = 17.3; 95% CI = 5.04, 59.34 for cigars; and RRR = 7.30; 95% CI = 2.04, 26.15 for hookah) as shown in Table 2.

**Table 2:** Association between experimentation and current tobacco use pattern among youth in the United States (2017).

	Exclusive ENDS users	Exclusive combustible mono-users	ENDS plus combustible poly-users	Poly combustible only users	Other poly tobacco users^a^
	RRR (95% CI)	RRR (95% CI)	RRR (95% CI)	RRR (95% CI)	RRR (95% CI)
**First tobacco tried**					
ENDS	Ref	Ref	Ref	Ref	Ref
Cigarettes	0.46 (0.28, 0.73)***	6.90 (2.62, 18.19)***	2.95 (1.90, 4.59)***	5.15 (1.92, 13.81)***	8.63 (3.64, 20.47)***
Cigar Group	0.32 (0.13, 0.81)*	17.3 (5.04, 59.3)***	4.32 (2.23, 8.38)***	5.78 (1.42, 23.53)*	16.95 (6.54, 43.95)***
Smokeless or Chewing	0.60 (0.20, 1.82)	0.18 (0.04, 0.91)*	0.10 (0.01, 0.66)*	2.38 (0.27, 20.83)	44.67 (15.38, 129.74)***
Hookah	0.33 (0.14, 0.79)*	7.30 (2.04, 26.15) **	5.03 (2.56, 9.89)***	4.65 (0.89, 24.26)	13.48 (4.5, 40.34)***
**Sex**					
Male	Ref	Ref	Ref	Ref	Ref
Female	0.52 (0.34, 0.81)**	0.90 (0.49, 1.66)	0.27 (0.18, 0.40)***	0.85 (0.39, 1.84)	0.07 (0.03, 0.16)***
**Sexual orientation**					
Heterosexual	Ref	Ref	Ref	Ref	Ref
LGB+	1.00 (0.60, 1.68)	1.11 (0.57, 2.13)	1.09 (0.69, 1.71)	1.06 (0.51, 2.18)	0.83 (0.38, 1.81)
**Age**					
13–14	Ref	Ref	Ref	Ref	Ref
15–16	0.97 (0.48, 1.94)	0.30 (0.10, 0.94)*	1.38 (0.72, 2.67)	0.31 (0.08, 1.19)	1.25 (0.63, 2.48)
17–18	1.43 (0.70, 2.93)	0.97 (0.36, 2.61)	2.74 (1.44, 5.22)**	0.98 (0.30, 3.20)	1.11 (0.56, 2.21)
**Race/ethnicity**					
NH-White	Ref	Ref	Ref	Ref	Ref
NH-Black	0.44 (0.24, 0.81)**	1.22 (0.60, 2.46)	0.65 (0.38, 1.12)	0.43 (0.10, 1.98)	0.33 (0.12, 0.91)*
Hispanic	0.65 (0.37, 1.13)	0.58 (0.24, 1.41)	0.66 (0.39, 1.12)	0.77 (0.29, 2.08)	0.81 (0.39, 1.67)
Other	0.84 (0.40, 1.78)	0.28 (0.06, 1.28)	0.79 (0.35, 1.81)	0.91 (0.15, 5.48)	1.19 (0.40, 3.52)
**Residence**					
Urban	Ref	Ref	Ref	Ref	Ref
Suburban	1.10 (0.71, 1.72)	0.54 (0.26, 1.12)	1.15 (0.75, 1.77)	0.53 (0.21, 1.37)	0.74 (0.42, 1.32)
Rural	0.85 (0.50, 1.44)	1.19 (0.62, 2.30)	0.56 (0.33, 0.97)*	0.96 (0.37, 2.46)	0.93 (0.45, 1.91)
**Public Assistance**					
Yes	Ref	Ref	Ref	Ref	Ref
No	1.14 (0.70, 1.85)	0.71 (0.39, 1.32)	1.03 (0.68, 1.56)	0.68 (0.32, 1.46)	0.52 (0.29, 0.95)*
Not Sure	0.51 (0.24, 1.05)	0.47 (0.17, 1.30)	0.55 (0.28, 1.07)	0.37 (0.09, 1.62)	0.08 (0.02, 0.29)***

ENDS: Electronic Nicotine Delivery Systems; Cigar Group: cigars, cigarillos, little cigars and bidis; RRR: adjusted relative risk ratio; CI: confidence interval

^a^Other poly tobacco users include all other current tobacco use combinations including combustible and/or non-combustible products, in addition to all exclusive smokeless and chewing current (n = 17) that was not included as self-standing group

*p<0.05;

**p<0.01;

***p<0.001

## Discussion

ENDS followed by combustible cigarettes were the most frequently reported first used tobacco products by our nationally representative sample of US youth. About 45% of youth experimenters become subsequent tobacco users. A notable finding is that poly tobacco use is becoming established among youth, accounting for more than 50% of the estimated use prevalence [10]. As anticipated, the subsequent pattern of tobacco use was notably linked to the initial tobacco product tried. Specifically, in our study, initial ENDS use was associated with a greater likelihood of being a subsequent exclusive ENDS user, and initial combustible experimentation was closely associated with subsequent combustible product use.

The evidence aligns with the expectation that individuals are inclined to persist with the first tobacco product they tried [23, 24]. Aligning with our hypothesis, the findings suggest that current tobacco users who initially experimented with a combustible tobacco product were more likely to be current combustible only or poly-tobacco users, and less likely to be exclusive ENDS users. Those who initially experiment with combustible products (cigarettes, cigars or hookah) seem likely to be youth who are either (a) high-risk for transitioning to poly-tobacco use or combustible tobacco products as part of their propensity for sensation-seeking and other risky behavior [25] or (b) prone to become more dependent on nicotine, which eventually leads to use of combustible or combustible plus ENDS products [10]. The initial experimentation phase may be of high importance to the youth tobacco experience and the resulting tobacco use pattern; youth who choose to initially experiment with a certain product (e.g., cigarettes) do not realistically consider its harms or addictive potential and may become dependent on this class of products [25]. More specifically, in our sample, half of the experimenters of non-ENDS products reported currently using tobacco in comparison to one-third of those who initially experimented with ENDS.

In the US, the majority of hookah users concurrently consume other tobacco products [23, 24, 26]. Those who used hookah first were more likely to ultimately be part of the ENDS plus combustible and other poly-tobacco users’ profiles rather than the exclusive combustible use group. Hookah use is a social experience, often taking place in hookah cafes, a setting that may be especially attractive to youth [27, 28]. However, the lack of portability of conventional hookah makes it challenging to sustain nicotine addiction from hookah alone. Thus, hookah may attract youth initially with its social appeal, with users then seeking more readily accessible products (e.g., cigarettes, ENDS) and becoming mixed (ENDS plus combustible) or poly-combustible tobacco users.

Finally, ENDS experimentation is associated with exclusive ENDS use rather than combustible or poly tobacco use. This finding could be interpreted in at least three ways. Perhaps those who experiment with ENDS may remain less dependent on nicotine and thus continue use of ENDS products that yield less nicotine than combustible cigarettes [10]. However, given the high nicotine content of popular pod-based products, such as late-generation ENDS or JUUL, this interpretation seems unlikely [29]. Another possibility is that ENDS users are mainly attracted to flavors and thus continue ENDS use exclusively, rather than switching to or adding combustible products without the variety of attractive flavors [30]. Alternately, as noted in previous work, perhaps youth perceive ENDS as less harmful than combustible tobacco; [31, 32] in other words, lower risk-oriented youth may be less likely to progress to tobacco-related behavior that they perceive as riskier (i.e., concomitant use of combustible or poly-tobacco use) [5]. This study cannot further test the latter hypothesis due to the cross-sectional nature of the data. Nevertheless, we divided the current tobacco use groups by exclusive or poly-use of products across all groups, and ENDS experimenters seemed to have the lowest likelihood for possessing poly-tobacco use profiles. But, given the low prevalence of non-combustible product use other than ENDS, we were not able to conduct necessary statistical analyses to determine predictors of membership for this tobacco use group independently, and there may be specific aspects that are not accounted for when incorporating these participants into the all-other tobacco use profile. Further, being on public assistance and from a rural area was inversely associated with using protobacco use, which may reflect the importance of market availability and affordability of tobacco products among youth. There is mixed evidence regarding ENDS serving as a gateway to subsequent use of other tobacco products, especially cigarettes [33, 34]. Nevertheless, it seems that initial experimentation with any tobacco product is important in shaping the subsequent tobacco use profile of youth.

A recent study found that youth experimenting with a new tobacco product can lead to various outcomes. Over time, users perceive the product as less harmful, and this experience influences their perceptions of harm for other tobacco products. Additionally, trying a new tobacco item increases susceptibility to experimenting with different tobacco products. These tendencies are especially notable among youth, indicating a complex interplay between experimentation, evolving perceptions, and heightened openness to using various tobacco products [35]. Although these connections have been consistently reproduced in existing literature, there hasn’t been an exhaustive exploration of empirical data to thoroughly investigate the potential mechanisms behind the longitudinal shifts in tobacco use among youth. The most recent study on NYTS survey demonstrated that, despite advancements, elements fostering tobacco product use among U.S. youths, such as the presence of flavored options, accessibility to tobacco products, exposure to marketing, and misconceptions about the risks associated with tobacco product use, remained widespread in 2021 among US youth [36].

The cross-sectional study design predisposes the findings to certain limitations. No causal statements can be made about the observed associations. We also included the small number of exclusive chew and smokeless dip users within the other tobacco use profile, which was mainly poly-tobacco users, instead of having a dedicated group, given their low prevalence. Caution is needed in interpreting those RRRs with wide confidence intervals due to small sample size and thus greater uncertainty. Data were self-reported; therefore, answers could have been affected by recall bias. This survey was also conducted prior to the FDA approval of heat-not-burn products in the US market in April 2019 [37], thus such products were not included in our study. In addition, some other aspects may have impacted our findings such as the short duration of the study that may preclude the authors from fully assessing the transition from experimenting phase to current use phase. Lastly, other potentially influential factors, such as parental tobacco use, age of first tobacco experimentation and level of nicotine dependence, were not accounted for in the analysis. Future longitudinal research could appraise the temporality of the association between first tobacco product used and transition to subsequent tobacco use.

The current findings can inform primary prevention activities. Given that youth seem to develop a preference for the tobacco product they first experiment with [23, 24], the tobacco control community needs to carefully consider the context and content of primary prevention efforts that are communicated to youth. Collectively, initial experimentation with tobacco products other than ENDS seemed to be significantly associated with continued use of tobacco products, suggesting that a higher proportion of initial ENDS experimenters (particularly from early cohorts potentially driven by the novelty of these products, i.e., early adopters) do not subsequently become current tobacco users [5]. Other than considering the main mandate of deterring youth from experimenting with any tobacco product, specific prevention messages could target those who tried specific tobacco products, with a focus on preventing them from moving to continued use of tobacco. These efforts could potentially deter lower risk-oriented youth from continuing to experiment with other tobacco products or becoming established tobacco users. Finally, tobacco control advocates should convey clearer assessment of the health implications of specific tobacco products to youth, rather than adopt a generalized less-risky message (e.g., comparison to combustible cigarettes, potential for harm reduction) that may result in youth perceiving ENDS as safer products [6].

## Conclusions

Prior research suggested that the pattern of tobacco use among youth was driven by their nicotine addiction [10]. Our findings suggest an additional perspective: The experience of initial experimentation with tobacco products may play a role in developing subsequent current tobacco use patterns among youth. Understanding the reasons behind initial product choice may help in understanding the reasons for subsequent current tobacco product use or the transition in patterns from combustible to non-combustible tobacco products or vice versa. Regulatory authorities and health campaign messaging should convey the risk of specific products to help prevent youth from experimenting with products they may consider safer options. Further understanding the reasons behind initial product choice for experimenting may help in understanding the reasons for subsequent current tobacco product use, thus informing youth prevention efforts.
